# Immunomodulatory Potential of 6-Gingerol and 6-Shogaol in *Lactobacillus plantarum*-Fermented *Zingiber officinale* Extract on Murine Macrophages

**DOI:** 10.3390/ijms26052159

**Published:** 2025-02-27

**Authors:** Ji Eun Kim, Kwang-Hyun Park, Jinny Park, Byeong Soo Kim, Geun-Seop Kim, Dong Geon Hwang

**Affiliations:** 1 Department of Companion and Laboratory Animal and Science, and Leaders in INdustryuniversity Cooperation 3.0 (LINC 3.0) Project by Ministry of Education, Kongju National University, Yesan 32439, Republic of Korea; wldms990218@naver.com (J.E.K.); bluenature26@gmail.com (G.-S.K.);; 2 Department of Emergency Medical Rescue, Nambu University, Gwangju 62271, Republic of Korea; khpark@nambu.ac.kr; 3 BioMedical Science Graduate Program (BMSGP), Chonnam National University, Hwasun 58128, Republic of Korea; 4 Department of Medical Oncology and Hematology, Ansan Hospital, Korea University College of Medicine, Ansan 15355, Republic of Korea

**Keywords:** *Zingiber officinale*, *Lactobacillus plantarum*, 6-gingerol, 6-shogaol, anti-inflammatory mechanism

## Abstract

In this study, we aimed to investigate whether the physiological activity of ethanol extracts of *Zingiber officinale* was improved after fermentation with *Lactobacillus plantarum* strains KCTC 3108 (FLP8) and KCL005 (FLP9). Total polyphenol and flavonoid content was substantially increased after fermentation with FLP8 and FLP9 for 48 h and 24 h, respectively, compared with the unfermented control. The 6-gingerol content was significantly increased in FLP9 after 24 h of fermentation, whereas in FLP8, it remained comparable to pre-fermentation levels. The 6-shogaol content significantly increased in FLP8 and FLP9 at 48 h and 24 h, respectively, compared with the pre-fermentation levels. The anti-inflammatory effects were evaluated using RAW 264.7 cells stimulated with lipopolysaccharides. The fermented product of FLP8 at 48 h and FLP9 at 24 h maintained over 80% cell viability at a concentration of 200 µg/mL and significantly reduced nitric oxide production compared to the lipopolysaccharide-stimulated control. Moreover, each extract downregulated pro-inflammatory gene expression. Furthermore, the purified 6-gingerol and 6-shogaol, which were purchased as reference compounds, were included in the fermentation extracts of FLP8 at 48 h and FLP9 at 24 h, and both inhibited cell migration in a dose-dependent manner without any cytotoxicity. In conclusion, the fermentation of *Z. officinale* with these *L. plantarum* strains enhanced its antioxidant and anti-inflammatory activities, with significant increases in bioactive compound content.

## 1. Introduction

*Zingiber officinale*, commonly known as ginger, is a perennial herbaceous plant belonging to the Zingiberaceae family native to subtropical and tropical regions. It is widely used as a spice owing to its unique flavor and aroma. The primary constituents of *Z. officinale* include carbohydrates (50–70%), lipids (3–8%), phenolic compounds, and 80–90% moisture [1]. *Z. officinale* is available in various forms, such as fresh and dried materials, oleoresins, and essential oils, and is used for culinary, medicinal, and cosmetic purposes [2]. It has been reported to possess various pharmacological activities, including anti-inflammatory, antioxidant, and anti-tumor properties [3]. Key bioactive components, such as zingiberene, γ-cardinen, zingiberol, gingerol, shogaol, and paradol, have been identified in *Z. officinale*. Among these, 6-gingerol is the predominant bioactive compound, exhibiting anti-inflammatory, analgesic, antipyretic, and antioxidant activities [2,3]. The dehydrated form of 6-gingerol, 6-shogaol, is known for its antimicrobial and antioxidant properties and is reported to have higher anti-inflammatory and antioxidant effects than 6-gingerol [4,5].

Fermentation is one of the oldest biotechnological processes used in food, medicine, and cosmetics. Various microbial starters, including *Lactobacillus* spp., *Bacillus* spp., *Pediococcus* spp., and *Bifidobacterium* spp., are used for fermentation [6]. The health benefits and functional properties of fermented foods, such as kimchi, jang, and jeotgal, have been recognized globally [7]. Probiotics, which are beneficial microorganisms used in fermentation, metabolize carbohydrates to produce bioactive substances and break down large molecules into smaller molecules, thereby improving digestion and absorption. Fermentation also enhances the flavor and aroma of foods, destroys toxic substances, improves digestibility, and increases the nutritional value by producing essential vitamins [8,9,10]. Organic acids produced by fermenting microorganisms inhibit the growth of spoilage bacteria, thereby enhancing the shelf life and safety of fermented foods [6,7].

Probiotics, primarily *Bifidobacterium* spp. and *Lactobacillus* spp., are live microorganisms that confer health benefits by maintaining or improving the gut microbial balance [11,12]. Lactic acid bacteria are Gram-positive, facultatively anaerobic bacteria that offer various health benefits, including the regulation of gut flora, reduction in serum cholesterol levels, and enhancement of the immune system [13]. These bacteria produce organic acids, such as lactic, acetic, and propionic acids, during fermentation, lowering the pH and secreting bacteriocins to inhibit foodborne pathogens and prevent spoilage [14]. They also exhibit antidiarrheal, cholesterol-lowering, anticancer, and immune-boosting effects [15,16].

Inflammatory responses are mediated by the activation of macrophages, which recognize lipopolysaccharides (LPSs) from bacterial cell walls through Toll-like receptor 4 (TLR4), leading to the activation of nuclear factor-κB (NF-κB) [17,18]. Activated macrophages produce inflammatory cytokines, including tumor necrosis factor (TNF-α), interleukin (IL)-6, and nitric oxide (NO) that contribute to various autoimmune diseases such as inflammatory bowel disease and rheumatoid arthritis [19,20]. The mitogen-activated protein kinase (MAPK) pathway, which includes extracellular signal-regulated kinase, p38, and c-Jun N-terminal kinase (JNK), is crucial for the regulation of pro-inflammatory cytokine activation [21]. The activation of macrophages via LPS-TLR4 interaction results in the phosphorylation of MAPK, leading to the activation of activator protein 1 and NF-κB, thereby inducing cytokine secretion [22,23]. Macrophages play a vital role in managing immune responses by overproducing pro-inflammatory cytokines such as TNF-α [24,25,26]. Cytokines produced by various cells, including macrophages and monocytes, are key regulators of inflammatory and allergic reactions [27,28].

This study compared the antioxidant and anti-inflammatory effects of *Z. officinale* before and after fermentation with *Lactobacillus plantarum* KCTC 3108 (FLP8) and *L. plantarum* KCL005 (FLP9). The objective was to develop a natural antioxidant and anti-inflammatory agent by maximizing these properties through fermentation.

## 2. Results

### 2.1. Fermentation Results and Analysis of Total Polyphenol and Flavonoid Content

**Figure 1:**
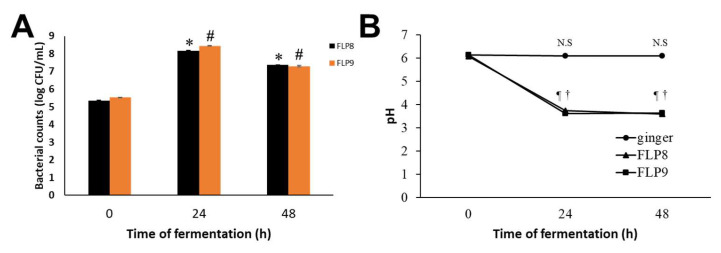
Growth profiles of FLP8 and FLP9 during *Zingiber officinale* extracts (ZOEs) fermentation and changes in pH of the fermenting medium. For the fermentation of ZOE, FLP8 or FLP9 were inoculated with 1% of FLPs Log CFU/mL changed during fermentation. Changes in (**A**) colony forming units (CFU)/mL of FLP8- and FLP9-fermented ZOE and (**B**) pH of cultures were measured at the times indicated. Data are represented as the mean ± SD. * *p* < 0.001 and # *p* < 0.001 vs. zero time group of each time point., N.S: Not significant, ¶ *p* < 0.001 and † *p* < 0.001 vs. zero time group of each time point FLP8 (48 h).

After fermentation of ZOE, the bacterial count (Log CFU/mL) of FLP8 increased from 5.34 ± 0.03 at 0 h to a peak of 8.17 ± 0.04 at 24 h, then slightly decreased to 7.37 ± 0.01 at 48 h. Similarly, the bacterial count of FLP9 increased from 5.55 ± 0.002 at 0 h to a peak of 8.45 ± 0.01 at 24 h, followed by a slight decline to 7.29 ±0.04 at 48 h (Figure 1A). The pH of unfermented ZOE was 6.13 ± 0.01, 6.11 ± 0.01, and 6.09 ± 0.01 at 0, 24, and 48 h, respectively. However, the pH of ZOE fermented with FLP8 and FLP9 markedly decreased to 3.74 ± 0.01 and 3.62 ± 0.01, respectively, at 24 h and showed no further significant change at 48 h (Figure 1B). The final recovery rate of the ethanol extract after fermentation with 100 g of ZOE was approximately 5.23% (freeze-dried powder, *w*/*w*).

**Figure 2:**
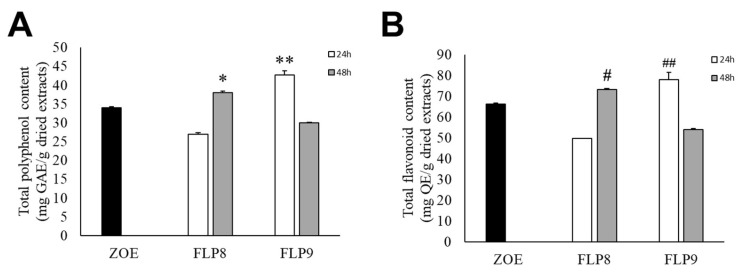
Effect on (**A**) total polyphenol and (**B**) flavonoid contents in ZOE fermented with FLP8 and FLP9. Extracts were fermented for the indicated times with each strain and analyzed for contents as described in the Materials and Methods Section. * *p* < 0.01 and ** *p* < 0.005 vs. ZOE control group; # *p* < 0.01, and ## *p* < 0.005 vs. ZOE control group.

The total polyphenol content of fermented ZOE was measured according to the fermentation time of each strain for 48 h at 37 °C (Figure 2A). The initial total polyphenol content in the ZOE prior to fermentation was 34.06 ± 0.15 mg/g. The total polyphenol content for ZOE fermented with FLP8 decreased to 27.00 ± 0.30 mg/g at 24 h but increased to 37.99 ± 0.38 mg/g at 48 h. After fermentation with FLP9, the polyphenol content increased to 42.71 ± 1.08 mg/g at 24 h but decreased to 29.91 ± 0.19 mg/g at 48 h. There was a significant increase in the total polyphenol content in the 48 h FLP8 group (FLP8 (48 h)) and the 24 h FLP9 group (FLP9 (24 h)) compared to the pre-fermentation levels. The total flavonoid content of fermented ZOE was also measured according to the fermentation time of each strain for 48 h at 37 °C (Figure 2B). Prior to fermentation, the initial total flavonoid content was 66.09 ± 0.46 mg/g. The flavonoid content for ZOE fermented with FLP8 decreased at 24 h compared to the pre-fermentation level, then increased to 73.20 ± 0.46 mg/g at 48 h. Similarly, for strain FLP9, the flavonoid content increased to 77.94 ± 3.56 mg/g at 24 h and decreased at 48 h. The 24 h FLP9 fermentation resulted in a significant increase in the total flavonoid content compared to the pre-fermentation level.

### 2.2. Changes in 6-Gingerol and 6-Shogaol Content

**Figure 3:**
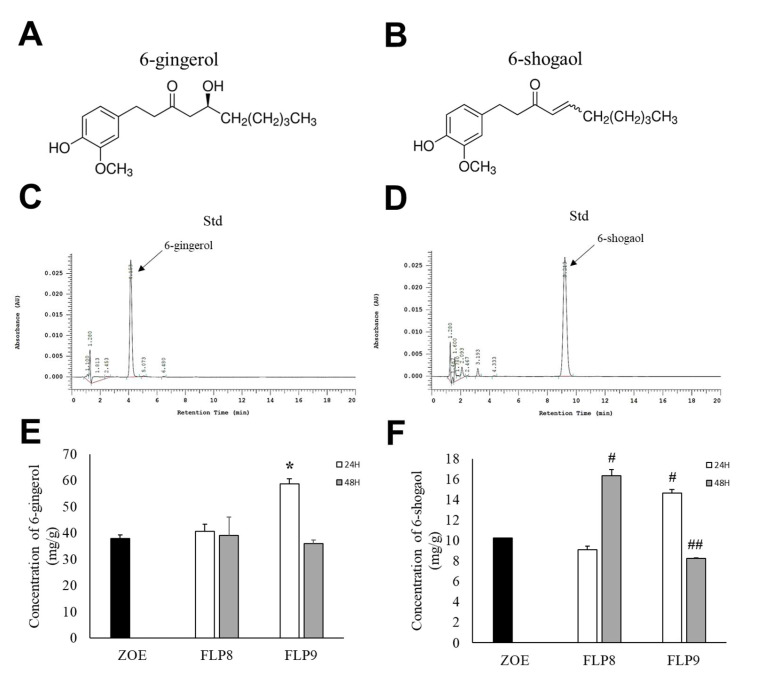
Effect on 6-gingerol and 6-shogaol contents in ZOE fermented by FLP8 and FLP9. Chemical structure of (**A**) 6-gingerol and (**B**) 6-shogaol. The representative standard peaks for each commercial standard compound for (**C**) 6-gingerol and (**D**) 6-shogaol were obtained. Differences in the levels of (**E**) 6-gingerol and (**F**) 6-shogaol were analyzed at each time point. Statistical analysis was performed using Student’s *t*-test, and data were obtained from three independent repetitions. * *p* < 0.001 vs. ZOE control group. # *p* < 0.001 and ## *p* < 0.05 vs. ZOE control group.

High-performance liquid chromatography (HPLC) was used to determine changes in 6-gingerol (Figure 3A) and 6-shogaol (Figure 3B) content after fermentation, wherein the difference between the two compounds arises from the presence or absence of a hydroxyl group (OH) at the respective positions. The retention times for 6-gingerol (Figure 3C) and 6-shogaol (Figure 3D) were 4.153 min and 9.213 min, respectively. The 6-gingerol content increased significantly by 54.33% in the FLP9 (24 h) group compared to that before fermentation and then decreased in the FLP9 (48 h) group to a level similar to that of the unfermented ZOE. In the FLP8 groups, the 6-gingerol content remained similar to that of the pre-fermented ZOE, and no significant changes were observed (Figure 3E). The 6-shogaol content significantly increased by 59.7% and 42.82% in the FLP8 (48 h) and FLP9 (24 h) groups, respectively, compared to that before fermentation (Figure 3F).

### 2.3. Endogenous Cytotoxicity and Inhibition of NO Production by Fermented ZOE in RAW 264.7 Cells

**Figure 4:**
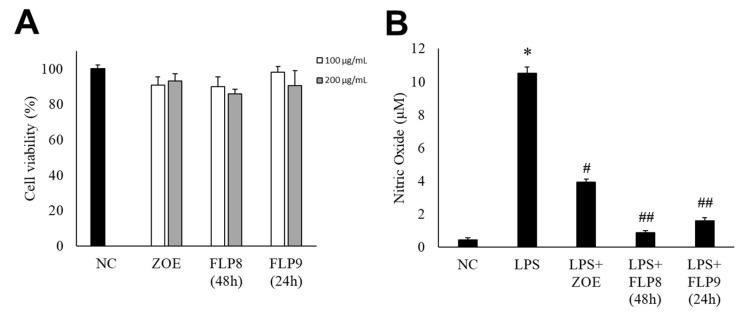
Endogenous cytotoxicity (**A**) and inhibitory effects on nitric oxide (NO) production in RAW 264.7 cells (**B**) by ZOE, FLP8 (48 h), and FLP9 (24 h). Cell viability was maintained above 80%, indicating that FLP8 (48 h) and FLP9 (24 h) had low cytotoxicity. Typical LPS-induced NO production was significantly reduced by FLP8 (48 h) and FLP9 (24 h) at a concentration of 200 µg/mL after 24 h of incubation. NC: Normal control group. Statistical analysis was performed using Student’s *t*-test, and data were obtained from three independent repetitions. * *p* < 0.001 vs. negative control group. # *p* < 0.005 and ## *p* < 0.001 vs. LPS only group.

The cytotoxicity of the fermented products of FLP8 (48 h) and FLP9 (24 h) was evaluated using the 3-(4,5-dimethylthiazol-2-yl)-2,5-diphenyltetrazolium bromide (MTT) assay. RAW 264.7 cells were treated with the FLP8 (48 h) and FLP9 (24 h) at concentrations of 100 and 200 µg/mL for 24 h. The results indicated that cell viability was maintained above 80% in all experimental groups, suggesting that the fermented products exhibited low toxicity. Consequently, An FLP concentration of 200 µg/mL was selected for subsequent experiments (Figure 4A). FLP8 (48 h) and FLP9 (24 h) exhibited relatively low toxicity, with viability levels stabilized even at high concentrations in RAW 264.7 cells.

NO production was measured in RAW 264.7 cells stimulated with LPS. LPS-induced NO production was significantly reduced in the ZOE, FLP8 (48 h), and FLP9 (24 h) treatment groups compared to that in the group treated with LPS alone (Figure 4B). The LPS-only group produced 10.51 ± 0.37 µM NO. By contrast, the NO production decreased by 91.77% to 0.87 ± 0.12 µM in the group treated with the FLP8 (48 h). Similarly, the FLP9 (24 h) group demonstrated a significant reduction in NO production compared to the unfermented ZOE group. Treatment with ZOE also inhibited LPS-induced NO production; however, the efficacy of FLP8 (48 h) and FLP9 (24 h) was greater.

### 2.4. Inhibitory Effect of Fermented ZOE on Inflammatory Cytokine Expression

**Figure 5:**
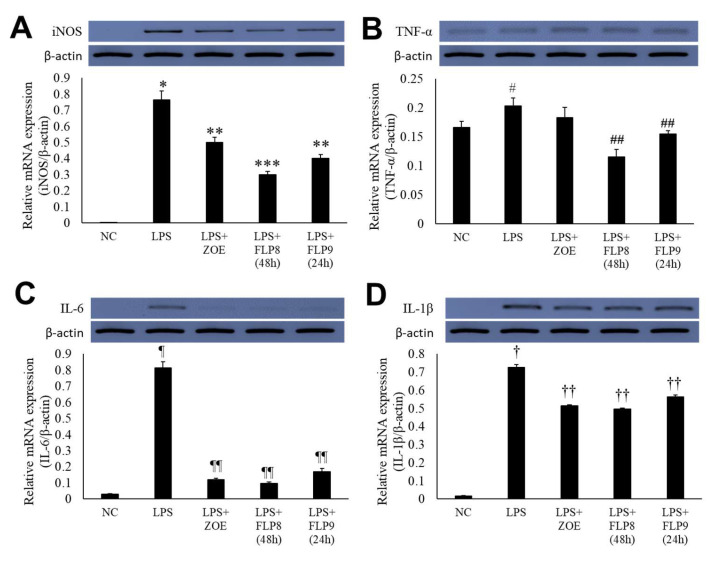
Inhibitory effect of fermented ZOE on inflammatory cytokine expression. Cells were stimulated by LPS in the presence or absence of ZOE, FLP8 (48 h), or FLP9 (24 h). The expression of (**A**) iNOS, (**B**) TNF-α, (**C**) IL-6, and (**D**) IL-1β mRNA levels were determined by RT-PCR. mRNA expression levels were quantified and statistically analyzed using ImageJ version 1.46 (NIH, Bethesda, MD, USA). * *p* < 0.001 vs. normal control (NC) group and ** *p* < 0.01, *** *p* < 0.005 vs. LPS only group. # *p* < 0.05 vs. NC group and ## *p* < 0.01 vs. LPS only group. Statistical analysis was performed using Student’s *t*-test, and data were obtained from three independent repetitions. Representative data exhibiting typical characteristics are presented. ¶ *p* < 0.001 vs. NC group and ¶¶ *p* < 0.005 vs. LPS only group. † *p* < 0.001 vs. NC group. †† *p* < 0.05 vs. LPS only group.

Changes in the expression of iNOS, TNF-α, IL-6, and IL-1β were assessed using RT-PCR (Figure 5A–D). In the LPS-only treatment group, gene expression of iNOS (Figure 5A), TNF-α (Figure 5B), IL-6 (Figure 5C), and IL-1β (Figure 5D) was markedly increased. However, treatment with ZOE, FLP8 (48 h), or FLP9 (24 h) for 24 h resulted in significant suppression of iNOS, IL-6, and IL-1β expression compared to the LPS-only group. Notably, the FLP8 (48 h) treatment significantly suppressed the mRNA levels of iNOS, TNF-α, IL-6, and IL-1β compared to unfermented ZOE. The expression of TNF-α, a key mediator of the LPS response and an indicator of innate immune response activation, was downregulated by 43.1% and 23.9% in the FLP8 (48 h) and FLP9 (24 h) groups, respectively, relative to the LPS-only treatment group. There was no significant difference between the ZOE and LPS-only treatment groups. The FLP8 (48 h) group demonstrated a pronounced inhibitory effect on the expression of inflammatory cytokines, exhibiting superior anti-inflammatory properties compared with unfermented ZOE.

### 2.5. Effects of 6-Gingerol and 6-Shogaol on Cell Migration in RAW 264.7 Cells

**Figure 6:**
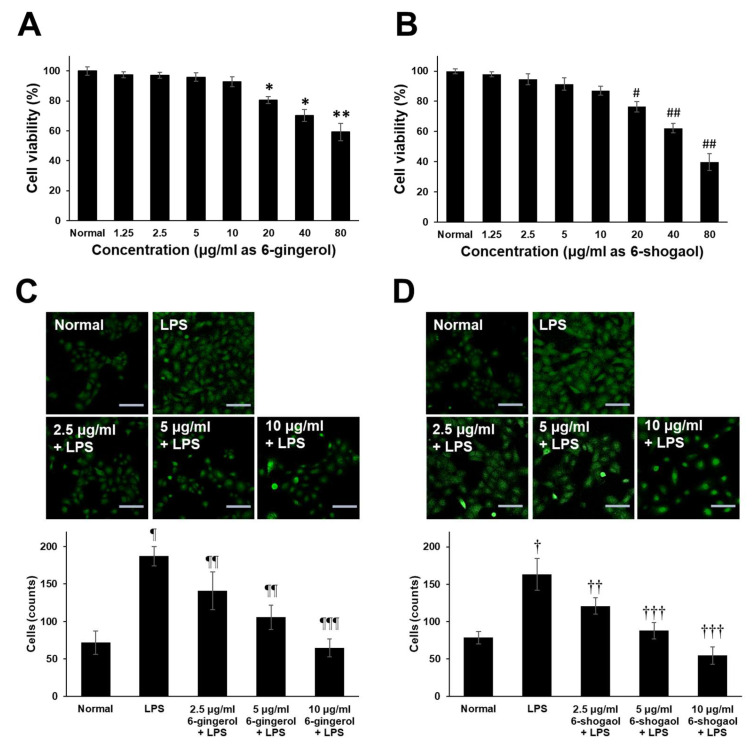
Endogenous cytotoxicity of 6-gingerol and 6-shogaol and effects on LPS-induced RAW 264.7 cells migration. Cells (5 × 10^5^ cells/mL) were cultured with different concentrations of (**A**) 6-gingerol and (**B**) 6-Shogaol (1.25–80 μg/mL) for 20 h. The cell viability was determined by MTT assay. Transwell migration assay of RAW 264.7 cells activated with LPS. Dose-dependent effect of different concentrations of (**C**) 6-gingerol and (**D**) 6-shogaol on RAW 264.7 cell migration was assessed using transwell migration assays over 20 h. Migrated and attached cells on the coverslip in the lower chamber were stained with anti-CD45-FITC. The upper panel shows representative fluorescence-positive cells (indicated as CD45-positive cells), and the lower panel presents the statistical analysis. Magnification = ×200. Scale bar = 100 μm. * *p* < 0.05, ** *p* < 0.01, # *p* < 0.01 and ## *p* < 0.005 vs. normal control goup. ¶ *p* < 0.01 and † *p* < 0.01 vs. normal control group. ¶¶ *p* < 0.05, ¶¶¶ *p* < 0.005, †† *p* < 0.05 and ††† *p* < 0.001 vs. LPS only group.

For the experimental assessment of the immunomodulatory potential of guaranteed 6-gingerol and 6-shogaol compounds which are present in the ZOE, FLP8 (48 h), and FLP9(24 h). This approach allowed for accurate quantification and direct evaluation of their effects on macrophage activity, providing a controlled setting to explore their role in immune modulation. To accurately assess the biological activity, we used commercially available purified 6-gingerol and 6-shogaol, thereby eliminating the potential interference of other compounds that could have been present in the fermented extracts. This approach minimizes the risk of inaccurate functional evaluation and provides a clearer understanding of the specific contributions of these compounds to the observed effects. The results demonstrated that both 6-gingerol and 6-shogaol significantly reduced the production of pro-inflammatory cytokines, including iNOS, TNF-α, IL-6, and IL-1β, in LPS-stimulated RAW 264.7 cells. The cytotoxicity of 6-gingerol and 6-shogaol was evaluated by culturing RAW 264.7 cells with various concentrations of these compounds (1.25–80 μg/mL) for 20 h. Both compounds exhibited low cytotoxicity across the tested concentrations (Figure 6A,B). Furthermore, treatment with 6-gingerol (Figure 6C) and 6-shogaol (Figure 6D) resulted in a dose-dependent inhibition of cell migration. Fluorescence staining with anti-CD45 antibody and subsequent quantification of migrated cells revealed a significant reduction in migration for both compounds in a dose-dependent manner, compared to the LPS-only group. These findings suggest that 6-gingerol and 6-shogaol, components of the fermented *Zingiber officinale* extract, modulate macrophage responses and may contribute to the regulation of inflammatory processes.

## 3. Discussion

Recent advances in the development of functional foods have increasingly focused on harnessing the inherent properties of plants. Microbial fermentation is a highly effective method for breaking down organic compounds in plants, thereby enhancing nutrient absorption [29,30]. *Z. officinale* is known for its significant role as a natural antioxidant in various food products [31]. The key compounds of *Z. officinale*, gingerol, and shogaol exhibit antioxidative, anti-inflammatory, anticancer, and anti-ulcer properties [32]. Many studies have categorized the chemical composition of *Z. officinale*. Particularly, 194 volatile oils, 85 gingerols, and 28 diarylheptanoid compounds have been classified [33], and the detailed mechanisms of specific compounds have been evaluated by molecular docking and dynamic studies in vitro [34]. Further advancements in new cultivation techniques, industrial applications, phytochemical constituent analysis, and investigation of the biological activities of *Zingiber* spp. are required [35].

In this study, we fermented ZOE with FLP8 and FLP9 and evaluated the antioxidative and anti-inflammatory effects of the fermentation products at various times. The fermentation conditions for FLP8 and FLP9 were optimized at different culture times (24 h and 48 h) and validated using well-known biological markers, including total polyphenols and total flavonoids [36,37]. Additionally, antioxidative effects of the fermentation were measured using DPPH and ABTS radical scavenging assays; however, no significant changes were observed between each experimental group, suggesting that these methods may not capture the subtle changes in antioxidative activity resulting from fermentation. We quantified the content of the functional, active components of *Z. officinale*, 6-gingerol, and 6-shogaol using HPLC and observed that both increased but at different times in fermentation. Some studies have suggested that fermentation conditions and microbial strains have a significant effect on the levels of these compounds [38,39], such as the manufacturing, processing, and storage of *Z. officinale* raw materials owing to dehydration and retroaldol reactions [40,41]. FLP8 and FLP9 showed relatively low cytotoxicity (>80% viability) at high concentrations (200 µg/mL), and the lowest NO production was recorded for FLP8 (48 h) and FLP9 (24 h). Maintenance of cell viability above 80% supports the conclusion that these materials are less cytotoxic. A potential explanation for this low toxicity may be that the endogenous native compounds in ZOE are transformed into less toxic forms. Therefore, specific fermentation of ZOE by each probiotic might enhance its regulatory effects against infection-related immune responses through the elevation of functional compound content. However, this may not guarantee effects on various human disorders owing to the presence of other components in pre- or post-processed materials.

FLP8 (48 h) and FLP9 (24 h) reduced the expression of LPS-induced inflammatory cytokine genes, including iNOS, TNF-ɑ, IL-6, and IL-1β; suggesting that 6-gingerol or other compounds may inhibit the expression of iNOS and ROS through PKC-ɑ and NF-κB [42]. FLP8 (48 h) reduced the phosphorylation of p44 and JNK in the MAPK signaling pathway, as well as the phosphorylation of p65 and IκB in the NF-κB signaling pathway. This finding is consistent with other studies demonstrating the inhibition of NF-κB, MAPK, and PI3K-Akt signaling pathways, thereby suppressing inflammation [43]. Additionally, 6-gingerol or 6-shogaol have been shown to exhibit anti-inflammatory effects [32,44,45]. The findings of this study highlight the immunomodulatory effects of 6-gingerol and 6-shogaol, key bioactive compounds present in fermented ZOE.

To enhance the interpretation of the immunomodulatory effects observed in our study, it is essential to consider the contributions of other bioactive compounds in the fermented ZOE, along with their chromatographic profiles. Previous studies have used chromatographic techniques such as HPLC and GC-MS to identify and quantify bioactive compounds in ginger [2,46,47,48], including gingerol, shogaol, paradol, and zingerone [4,5,39]. For instance, HPLC analysis has shown specific retention times for 6-gingerol (4.153 min) and 6-shogaol (9.213 min), which correlate with their bioactivity. These profiles provide insight into the composition of the extract and changes during fermentation. Chromatographic data indicate that fermentation can alter the levels of compounds like 6-gingerol and 6-shogaol, which increase during fermentation and contribute to enhanced anti-inflammatory effects. GC-MS profiles have also revealed changes in the concentration of volatile oils and diarylheptanoids [33,48] associated with anti-inflammatory activity. These findings underscore the dynamic nature of fermentation, influencing the final bioactivity of the extract. Therefore, it is important to consider chromatographic profiles and the variations in these compounds to fully understand the immunomodulatory effects of fermented *Z. officinale*.

The observed reduction in pro-inflammatory cytokine expression, including iNOS, TNF-α, IL-6, and IL-1β, suggests that these compounds play a significant role in attenuating macrophage-mediated inflammatory responses. Notably, both 6-gingerol and 6-shogaol demonstrated low cytotoxicity across a wide concentration range and exhibited a dose-dependent inhibition of macrophage migration while showing the potential to regulate immune cell mobility during inflammation. These results collectively suggest that the fermentation process not only enhances the bioavailability of 6-gingerol and 6-shogaol but also amplifies their biological activity. Consequently, the immunomodulatory effects observed in this study are largely attributable to these bioactive components within the fermented extract, positioning them as promising candidates for managing inflammatory conditions [49,50,51].

Our study suggests that FLP8 (48 h) and FLP9 (24 h) fermentation of ZOE enhanced anti-inflammatory functions compared to the unfermented form. This improvement can be attributed to the fermentation process, which appears to increase the bioavailability of active compounds, including 6-shogaol and 6-gingerol. These findings also indicate that microbial fermentation improves the functional properties of ZOE components. Therefore, fermented products are promising natural functional food ingredients with potential applications in the management of inflammation-related conditions. However, our study lacked in vivo experiments to confirm the anti-inflammatory effects observed in vitro. Additionally, the precise mechanisms by which fermentation enhances the bioavailability of active compounds remain unclear; therefore, further investigation is required using various disease models.

This study suggests that fermenting ZOE with FLP8 and FLP9 enhances the total polyphenol and flavonoid content, antioxidative potential, and expression of inflammatory cytokine genes compared to unfermented materials. This reflects the broader impact of fermentation on the bioactive properties of the extract. Interestingly, FLP8 (48 h) and FLP9 (24 h) consistently exhibited superior antioxidant and anti-inflammatory properties. The FLP8 (48 h) and FLP9 (24 h) fermentation of ZOE exhibited anti-inflammatory effects through the inhibition of MAPK and NF-κB signaling phosphorylation, suggesting their potential utility in the prevention and treatment of inflammatory diseases. This study highlights the potential of fermented ZOE as a natural functional food ingredient and provides a valuable foundation for future research and clinical applications.

## 4. Materials and Methods

### 4.1. Materials and Reagents

*Z. officinale* was harvested from Yesan-eup (Yesan-gun, Chungcheongnam-do, Republic of Korea), and the rhizome peels were meticulously removed. *L. plantarum* KCTC 3108 (FLP8) was obtained from the Biological Resource Center of the Korea Research Institute of Bioscience and Biotechnology (KCTC, KRIBB, Daejeon, Republic of Korea). The *L. plantarum* KCL005 (FLP9) strain was selected from the distributed strains for its high antioxidant activity [52]. All strains were cultured in Lactobacilli MRS broth (MRS, Difco, Detroit, MI, USA) at 37 °C for 24 h. Post-cultivation, the bacterial cultures were stored with 15% glycerol at −70 °C for future use.

### 4.2. Fermentation and Extraction

The raw *Z. officinale* solids were incorporated to obtain a 20% (*w*/*v*) concentration, homogenized with ice-cold distilled water using a laboratory blender (Waring Laboratory Science, Stamford, CT, USA). The *Z. officinale* mixture was filtered through a 100-mesh filter (Millipore, Burlington, MA, USA) under aseptic conditions at room temperature. FLP8 and FLP9 were pre-cultured in MRS broth (Difco, Detroit, MI, USA) at 37 °C for 24 h. For fermentation, the ZOE was inoculated with pre-cultured FLP8 or FLP9, each adjusted to a McFarland standard of 0.5, at a concentration of 1% (*v*/*v*), then further incubated statically at 37 °C for 48 h in dark conditions. Samples of fermented ZOE were collected at 0, 24, and 48 h to measure the CFU of the bacteria and pH. The samples were diluted in peptone water and inoculated onto MRS agar plates, followed by incubation at 37 °C for 24 h before measuring the CFU. The pH of the fermentation samples was measured using a pH meter. The remaining samples were subsequently stored at −70 °C for further analysis. For extraction, 100 g of the fermented ZOE sample slurry was mixed with 1 L of 95% (*v*/*v*) ethanol and sonicated for 2 h. The extracted fermented material was then concentrated using a rotary evaporator (Hanil Scientifics, Seoul, Republic of Korea). The resulting freeze-dried powder was stored at −80 °C and filtered through a 0.45 µm filter (Millipore, Burlington, MA, USA) prior to use.

### 4.3. Measurements of Total Polyphenol and Flavonoid Content

Non-flavonoid and flavonoid compounds containing total polyphenols [53,54] and flavonoids [53,55] in FLP8- and FLP9-fermented ZOE were measured using a methanolic solution of gallic acid and quercetin as standards. For the total polyphenol assay, 500 µL of Folin–Ciocalteu’s phenol reagent was added to each sample and incubated at room temperature for 3 min. Subsequently, 400 µL of 1 M Na_2_CO_3_ was added and further incubated for 1 h at room temperature. For the total flavonoids assay, 300 µL of 1 M NaNO_2_ was added to each sample and incubated at room temperature for 5 min. Subsequently, 300 µL of 10% AlCl_3_ was added and further incubated for 6 min at room temperature before adding 2 mL of 1 N NaOH and 1.9 mL of distilled water and incubating the mixture in the dark for 15 min. Absorbance was then measured at 750 nm and 510 nm for the total polyphenol and total flavonoid assays, respectively, using a microplate reader (Biotek, Seoul, Republic of Korea).

### 4.4. Analysis of 6-Gingerol and 6-Shogaol Contents

The 6-gingerol and 6-shogaol contents in the ZOE were analyzed using HPLC. The concentrated fermented extract was dissolved in methanol at a concentration of 0.4 g/mL and subjected to ultrasonic extraction for 1 h. 6-gingerol and 6-shogaol were quantified using standard calibration curves prepared under the conditions listed in Table 1.

### 4.5. Cell Culture and Cytotoxicity Assay

RAW 264.7 murine macrophage cells were purchased from the Korean Cell Line Bank (KCLB, Seoul, Republic of Korea). The cells were cultured in Dulbecco’s Modified Eagle Medium (DMEM) supplemented with 10% fetal bovine serum (Hyclone, Logan, UT, USA) and 1% antibiotic-antimycotic (Thermo Fisher Scientific, Waltham, MA, USA) at 37 °C in a 5% CO_2_ atmosphere.

The endogenous cytotoxicity of ZOE on RAW 264.7 cells was assessed using the MTT assay [56]. Cells were seeded in a 96-well plate at a density of 5 × 10^5^ cells/mL in DMEM and incubated at 37 °C in a 5% CO_2_ incubator for 24 h. The cells were then treated with fermented ZOE and incubated for a further 24 h. After the MTT assay, the resulting formazan crystals were dissolved in dimethyl sulfoxide (Daejung, Seoul, Republic of Korea), and the absorbance was measured at 570 nm.

### 4.6. Nitrite Assay

Cells were seeded in a 96-well plate at a density of 5 × 10^5^ cells/mL and incubated for 24 h. The medium was then removed, and the cells were washed thrice with phosphate-buffered saline (PBS). Fresh, ice-cold DMEM containing ZOE was then added and further incubated for 12 h. The medium was then removed, and the negative control group received only DMEM, whereas the other groups were treated with DMEM containing 100 ng/mL LPS. After 12 h of incubation, 200 µL of the supernatant was centrifuged at 3000 rpm for 2 min at 4 °C. Then, 100 µL of the supernatant was transferred to a new 96-well plate, and Griess reagent (Sigma, St. Louis, MO, USA) was added. The mixture was incubated at room temperature for 30 min, and the absorbance was measured at 548 nm (Biotek, Seoul, Republic of Korea).

### 4.7. RNA Extraction and RT-PCR

Cells were seeded in 6-well plates (SPL, Seoul, Republic of Korea) at a density of 3 × 10^5^ cells/mL and incubated for 24 h. After incubation, the cells were treated with a fresh, ice-cold medium containing the appropriate sample. The negative control group was treated with fresh medium, whereas the test groups were treated with 100 ng/mL LPS-containing medium and incubated for 12 h.

Cells were washed twice in 1 mL ice-cold PBS before transferring to a 1.8 mL microcentrifuge tube and centrifuging at 2000 rpm for 3 min. The pellet was resuspended in 1 mL TRIzol reagent (Sigma, St. Louis, MO, USA) and centrifuged at 12,395 rpm for 3 min. Total RNA was extracted and quantified using a spectrophotometer (Biotek, Seoul, Republic of Korea). For cDNA synthesis, 1 µg of RNA was reverse-transcribed using a cDNA synthesis kit (Thermo Fisher Scientific, USA), according to the manufacturer’s instructions. The primers used for PCR are listed in Table 2. Electrophoresis was performed on a 1.2% agarose gel at 100 V for 20 min.

### 4.8. In Vitro Migration Assay

Chemotactic migration of RAW 264.7 cells was analyzed using a modified Transwell^TM^ assay with cell culture membrane inserts with 8 μm pore size (Merck KGaA, Darmstadt, Germany) in 24 well plates. A total of 2 × 10^5^ cells were placed with experimental materials (LPS, 6-gingerol or 6-shogaol) in 0.5% FBS-DMEM in the upper chamber, while the culture medium in the lower compartment contained 10% FBS-DMEM with a sterilized 12 mm glass coverslip (Thermo Fisher Scientific Inc., MA, USA). After 20 h, non-migratory cells on top of the membrane chamber were removed, and the coverslips in the lower compartment were fixed with methanol for 10 s [57]. The transmigrated and attached cells on the coverslip were stained with Goat anti-CD45 antibody (AF114, R&D Systems, Minnneapolis, MN, USA) and anti-Goat IgG-FITC conjugates secondary antibody (Advanced Biochemicals Inc., Jeonju, Republic of Korea). For each mounted coverslip, 4–6 random fields were observed using an inverted Nikon Eclipse TE 2000-E confocal microscope system (Nikon, Tokyo, Japan), and the number of positively stained cells was counted [49,50,51].

### 4.9. Statistical Analysis

The experimental data were expressed as mean ± standard deviations (SD). Statistical analysis was performed using Student’s *t*-test, and a *p* value of <0.05 was considered statistically significant.

## Figures and Tables

**Table 1:** Instrumental operating conditions for high-performance liquid chromatography (HPLC).

Items	Condition
Instrument	Hitachi HPLC Primaide (Hitachi, Tokyo, Japan)
Column	Brownlee choice C18 (4.6 × 150 mm, 5 μm)
Column oven	30 °C
Detector	UV detector
Absorbance	282 nm
Flow rate	1.0 mL/min
Mobile phase	Isocratic (MeOH:D.W. = 80:20)
Injection volume	10 μL

**Table 2:** Specific primer sequences were used for RT-PCR.

Genes	Primer Sequences (5′ to 3′)
*iNOS*	F: TCTTTGACGCTCGGAACTGT R: CCATGATGGTCACATTCTGC
*TNF-α*	F: CACAAGATGCTGGGACAGTGA R: TCCTTGATGGTGGTGCATGA
*IL-6*	F: AGTTGCCTTCTTGGGACTGA R: CAGAATTGCCATTGCACAAC
*IL-1β*	F: GACCTTCCAGGATGAGGACA R: AGCTCATATGGGTCCGACAG
*β* *-actin*	F: CCTGAACCCTAAGGCCAACC R: CAGCTGTGGTGGTGAAGCTG

## Data Availability

Data is contained within the article.

## References

[1] Prasad S., Tyagi A.K. (2015). Ginger and Its Constituents: Role in Prevention and Treatment of Gastrointestinal Cancer. Gastroenterol. Res. Pract..

[2] Dara M.A., Mohammed A.W., Bnar M.I. (2015). Antimicrobial and antioxidant activities of extracts from medicinal plant ginger (*Zingiber officinale*) and identification of components by gas chromatography. Afr. J. Plant Sci..

[3] Mahomoodally M.F., Aumeeruddy M.Z., Rengasamy K.R.R., Roshan S., Hammad S., Pandohee J., Hu X., Zengin G. (2021). Ginger and its active compounds in cancer therapy: From folk uses to nano-therapeutic applications. Semin. Cancer Biol..

[4] Chen H., Fu J., Chen H., Hu Y., Soroka D.N., Prigge J.R., Schmidt E.E., Yan F., Major M.B., Chen X. (2014). Ginger Compound [6]-Shogaol and Its Cysteine-Conjugated Metabolite (M2) Activate Nrf2 in Colon Epithelial Cells in Vitro and in Vivo. Chem. Res. Toxicol..

[5] Ok S., Jeong W.-S. (2012). Optimization of Extraction Conditions for the 6-Shogaol-rich Extract from Ginger (*Zingiber officinale* Roscoe). Prev. Nutr. Food Sci..

[6] Kaur H., Kaur G., Ali S.A. (2022). Dairy-Based Probiotic-Fermented Functional Foods: An Update on Their Health-Promoting Properties. Fermentation.

[7] Marco M.L., Heeney D., Binda S., Cifelli C.J., Cotter P.D., Foligné B., Gänzle M., Kort R., Pasin G., Pihlanto A. (2017). Health benefits of fermented foods: Microbiota and beyond. Curr. Opin. Biotechnol..

[8] Sharma R., Garg P., Kumar P., Bhatia S.K., Kulshrestha S. (2020). Microbial Fermentation and Its Role in Quality Improvement of Fermented Foods. Fermentation.

[9] Voidarou C., Antoniadou M., Rozos G., Tzora A., Skoufos I., Varzakas T., Lagiou A., Bezirtzoglou E. (2021). Fermentative Foods: Microbiology, Biochemistry, Potential Human Health Benefits and Public Health Issues. Foods.

[10] Latif A., Shehzad A., Niazi S., Zahid A., Ashraf W., Iqbal M.W., Rehman A., Riaz T., Aadil R.M., Khan I.M. (2023). Probiotics: Mechanism of action, health benefits and their application in food industries. Front. Microbiol..

[11] Barzegar H., Alizadeh Behbahani B., Falah F. (2021). Safety, probiotic properties, antimicrobial activity, and technological performance of Lactobacillus strains isolated from Iranian raw milk cheeses. Food Sci. Nutr..

[12] Das T.K., Pradhan S., Chakrabarti S., Mondal K.C., Ghosh K. (2022). Current status of probiotic and related health benefits. Appl. Food Res..

[13] Kechagia M., Basoulis D., Konstantopoulou S., Dimitriadi D., Gyftopoulou K., Skarmoutsou N., Fakiri E.M. (2013). Health Benefits of Probiotics: A Review. Int. Sch. Res. Not..

[14] Peng W., Meng D., Yue T., Wang Z., Gao Z. (2020). Effect of the apple cultivar on cloudy apple juice fermented by a mixture of *Lactobacillus acidophilus*, *Lactobacillus plantarum*, and *Lactobacillus fermentum*. Food Chem..

[15] Lee J.M., Heo S.S. (2021). The Role of Probiotics in Human Health. J. Microbiol. Biotechnol..

[16] Terpou A., Papadaki A., Lappa I.K., Kachrimanidou V., Bosnea L.A., Kopsahelis N. (2019). Probiotics in Food Systems: Significance and Emerging Strategies Towards Improved Viability and Delivery of Enhanced Beneficial Value. Nutrients.

[17] Hoshino K., Takeuchi O., Kawai T., Sanjo H., Ogawa T., Takeda Y., Takeda K., Akira S. (2016). Pillars Article: Cutting Edge: Toll-Like Receptor 4 (TLR4)-Deficient Mice Are Hyporesponsive to Lipopolysaccharide: Evidence for TLR4 as the Lps Gene Product. J. Immunol..

[18] Kawai T., Akira S. (2010). The role of pattern-recognition receptors in innate immunity: Update on Toll-like receptors. Nat. Immunol..

[19] Chen S., Saeed A.F., Liu Q., Jiang Q., Xu H., Xiao G.G., Rao L., Duo Y. (2023). Macrophages in immunoregulation and therapeutics. Sig. Transduct Target Ther..

[20] Mosser D.M., Edwards J.P. (2008). Exploring the full spectrum of macrophage activation. Nat. Rev. Immunol..

[21] Bromberg J., Wang T.C. (2009). Inflammation and Cancer: IL-6 and STAT3 Complete the Link. Cancer Cell.

[22] Kawai T., Akira S. (2007). Signaling to NF-κB by Toll-like receptors. Trends Mol. Med..

[23] Liu T., Zhang L., Joo D., Sun S.-C. (2017). NF-κB signaling in inflammation. Signal Transduct. Target. Ther..

[24] De Paepe B., Creus K.K., De Bleecker J.L. (2009). Role of cytokines and chemokines in idiopathic inflammatory myopathies. Curr. Opin. Rheumatol..

[25] Vezza T., Rodríguez-Nogales A., Algieri F., Utrilla M.P., Rodriguez-Cabezas M.E., Galvez J. (2016). Flavonoids in Inflammatory Bowel Disease: A Review. Nutrients.

[26] Hegarty L.M., Jones G.-R., Bain C.C. (2023). Macrophages in intestinal homeostasis and inflammatory bowel disease. Nat. Rev. Gastroenterol. Hepatol..

[27] Duque G.A., Descoteaux A. (2014). Macrophage Cytokines: Involvement in Immunity and Infectious Diseases. Front. Immunol..

[28] Zhang J.M., An J. (2007). Cytokines, inflammation, and pain. Int. Anesthesiol. Clin..

[29] Liu M., Tian X., He L., Li C., Tao H., Wang X., Qiao S., Zeng X. (2023). Effects of tandem fermentation of edible mushroom and *L. plantarum* on sensory, polysaccharide, vitamin C, and γ-aminobutyric acid of *Rosa roxburghii Tratt* and coix seed beverage. Food Chem. X.

[30] Kim N.-M., Lee J.-S. (2003). Effect of Fermentation Periods on the Qualities and Physiological Functionalities of the Mushroom Fermentation Broth. Korean J. Mycol..

[31] Stoilova I., Krastanov A., Stoyanova A., Denev P., Gargova S. (2007). Antioxidant activity of a ginger extract (*Zingiber officinale*). Food Chem..

[32] Dugasani S., Pichika M.R., Nadarajah V.D., Balijepalli M.K., Tandra S., Korlakunta J.N. (2010). Comparative antioxidant and anti-inflammatory effects of [6]-gingerol, [8]-gingerol, [10]-gingerol and [6]-shogaol. J. Ethnopharmacol..

[33] Liu Y., Liu J., Zhang Y. (2019). Research Progress on Chemical Constituents of *Zingiber officinale* Roscoe. BioMed Res. Int..

[34] Rampogu S., Baek A., Gajula R.G., Zeb A., Bavi R.S., Kumar R., Kim Y., Kwon Y.J., Lee K.W. (2018). Ginger (*Zingiber officinale*) phytochemicals—Gingerenone-A and shogaol inhibit SaHPPK: Molecular docking, molecular dynamics simulations and in vitro approaches. Ann. Clin. Microbiol. Antimicrob..

[35] Sharifi-Rad M., Varoni E.M., Salehi B., Sharifi-Rad J., Matthews K.R., Ayatollahi S.A., Kobarfard F., Ibrahim S.A., Mnayer D., Zakaria Z.A. (2017). Plants of the Genus Zingiber as a Source of Bioactive Phytochemicals: From Tradition to Pharmacy. Molecules.

[36] Saleh R.M., Kabli S., Al-Garni S.M., Al-Ghamdi M., Abdel-Aty A.M., Mohamed S. (2018). Solid-state fermentation by Trichoderma viride for enhancing phenolic content, antioxidant and antimicrobial activities in ginger. Lett. Appl. Microbiol..

[37] Filailla E., Mulyani H., Maryati Y., Budiari S. (2022). The effect of fermentation time and inoculum amount on total sugar, to-tal acid, total flavonoid, total phenol, and inhibition of alpha-glucosidase activity of red ginger kombucha (*Zingiber officinale* Roscoe). J. Kim. Terap. Indones..

[38] Seo Y.-H. (2017). Antioxidant and antimicrobial activities of ginger with aging and fermentation. Korean J. Food Preserv..

[39] Choi J.W., Park H.-Y., Oh M.S., Yoo H.H., Lee S.-H., Ha S.K. (2017). Neuroprotective effect of 6-paradol enriched ginger extract by fermentation using *Schizosaccharomyces pombe*. J. Funct. Foods.

[40] Gao Y., Lu Y., Zhang N., Udenigwe C.C., Zhang Y., Fu Y. (2022). Preparation, pungency and bioactivity of gingerols from ginger (*Zingiber officinale* Roscoe): A review. Crit. Rev. Food Sci. Nutr..

[41] Lee H.-R., Lee J.-H., Park C.-S., Ra K.-R., Ha J.-S., Cha M.-H., Kim S.-N., Choi Y., Hwang J., Nam J.-S. (2014). Physicochemical Properties and Antioxidant Capacities of Different Parts of Ginger (*Zingiber officinale* Roscoe). J. Korean Soc. Food Sci. Nutr..

[42] Lee T.-Y., Lee K.-C., Chen S.-Y., Chang H.-H. (2009). 6-Gingerol inhibits ROS and iNOS through the suppression of PKC-α and NF-κB pathways in lipopolysaccharide-stimulated mouse macrophages. Biochem. Biophys. Res. Commun..

[43] Haque A., Jantan I., Harikrishnan H., Ghazalee S. (2019). Standardized extract of Zingiber zerumbet suppresses LPS-induced pro-inflammatory responses through NF-κB, MAPK and PI3K-Akt signaling pathways in U937 macrophages. Phytomedicine.

[44] Li F., Nitteranon V., Tang X., Liang J., Zhang G., Parkin K.L., Hu Q. (2012). In vitro antioxidant and anti-inflammatory activities of 1-dehydro-[6]-gingerdione, 6-shogaol, 6-dehydroshogaol and hexahydrocurcumin. Food Chem..

[45] Pan M., Hsieh M., Hsu P., Ho S., Lai C., Wu H., Sang S., Ho C. (2008). 6-Shogaol suppressed lipopolysaccharide-induced up-expression of iNOS and COX-2 in murine macrophages. Mol. Nutr. Food Res..

[46] You H., Ireland B., Moeszinger M., Zhang H., Snow L., Krepich S., Takagawa V. (2019). Determination of bioactive nonvolatile ginger constituents in dietary supplements by a rapid and economic HPLC method: Analytical method development and single-laboratory validation. Talanta.

[47] Rajput A.H., Gavali R.D., Jadhav A.P. (2023). Development and validation of a novel high-performance thin-layer chromatography method for the quantitative estimation of zingerone. JPC J. Planar Chromatogr. Mod. TLC.

[48] Yu Y., Huang T., Yang B., Liu X., Duan G. (2007). Development of gas chromatography–mass spectrometry with microwave distillation and simultaneous solid-phase microextraction for rapid determination of volatile constituents in ginger. J. Pharm. Biomed. Anal..

[49] Liu J., Pan T., You X., Xu Y., Liang J., Limpanont Y., Sun X., Okanurak K., Zheng H., Wu Z. (2015). SjCa8, a calcium-binding protein from Schistosoma japonicum, inhibits cell migration and suppresses nitric oxide release of RAW264.7 macrophages. Parasites Vectors.

[50] Bischoff-Kont I., Primke T., Niebergall L.S., Zech T., Fürst R. (2022). Ginger Constituent 6-Shogaol Inhibits Inflammation- and Angiogenesis-Related Cell Functions in Primary Human Endothelial Cells. Front. Pharmacol..

[51] Isa Y., Miyakawa Y., Yanagisawa M., Goto T., Kang M.-S., Kawada T., Morimitsu Y., Kubota K., Tsuda T. (2008). 6-Shogaol and 6-gingerol, the pungent of ginger, inhibit TNF-α mediated downregulation of adiponectin expression via different mechanisms in 3T3-L1 adipocytes. Biochem. Biophys. Res. Commun..

[52] Kim G.-S., Yang K.H., Kim H.-K., Kim J.-E., Yun H.-N., Yu J.-W., Kim B.-S. (2022). Antioxidant and Immunomodulatory Effect of Lactic Acid Bacteria Fermented Barley Sprout Hot Water Extract. J. Korean Soc. Food Sci. Nutr..

[53] Milella R.A., Basile T., Alba V., Gasparro M., Giannandrea M.A., DeBiase G., Genghi R., Antonacci D. (2019). Optimized ultrasonic-assisted extraction of phenolic antioxidants from grape (*Vitis vinifera* L.) skin using response surface methodology. J. Food Sci. Technol..

[54] Tutino V., Gigante I., Milella R.A., De Nunzio V., Flamini R., De Rosso M., Scavo M.P., Depalo N., Fanizza E., Caruso M.G. (2020). Flavonoid and Non-Flavonoid Compounds of Autumn Royal and Egnatia Grape Skin Extracts Affect Membrane PUFA’s Profile and Cell Morphology in Human Colon Cancer Cell Lines. Molecules.

[55] Pothitirat W., Chomnawang M.T., Supabphol R., Gritsanapan W. (2009). Comparison of bioactive compounds content, free radical scavenging and anti-acne inducing bacteria activities of extracts from the mangosteen fruit rind at two stages of maturity. Fitoterapia.

[56] Mosmann T. (1983). Rapid colorimetric assay for cellular growth and survival: Application to proliferation and cytotoxicity assays. J. Immunol. Methods.

[57] Müller S., Quast T., Schröder A., Hucke S., Klotz L., Jantsch J., Gerzer R., Hemmersbach R., Kolanus W. (2013). Salt-Dependent Chemotaxis of Macrophages. PLoS ONE.

